# Supplementary material for: Evaluation of the landscape of pharmacodynamic biomarkers in Niemann-Pick Disease Type C (NPC)
Source: Orphanet J Rare Dis. 2024 Jul 26;19:280. doi: 10.1186/s13023-024-03233-7 (PMC11282650; doi:10.1186/s13023-024-03233-7)
Supplement: Supplementary file 1 — Supplementary Material 1 [file 13023_2024_3233_MOESM1_ESM.pdf]

Evaluation of the Landscape of Pharmacodynamic Biomarkers in Niemann-Pick Disease Type C (NPC)

Sydney Stern[¥], Karryn Crisamore[¥], Robert Schuck, and Michael Pacanowski

¥ denotes equal contribution

Center for Drug Evaluation and Research, Office of Translational Science, Office of Clinical Pharmacology, US Food and Drug Administration, Silver Spring, Maryland (S.S., K.C., R.S., M.P.)

Running title: Pharmacodynamic Markers in NPC

Corresponding Authors: Sydney Stern, PhD, Office of Clinical Pharmacology, Office of Translational Science, Center for Drug Evaluation and Research, U.S. Food and Drug Administration, 10903 New Hampshire Ave, Silver Spring, Maryland 20993, USA; Phone: (301) 796-3919: Email: Sydney.Stern@fda.hhs.gov

Text pages: 38

Figures: 1

Tables: 1

References: 107

Number of words in Abstract: 200

Number of words in Introduction: 887

Number of words in Discussion: 1063

Number of words in Text: 7001

Abstract

Niemann-Pick disease Type C (NPC) is an autosomal recessive, progressive disorder resulting from variants in *NPC1* or *NPC2* that leads to the accumulation of cholesterol and other lipids in the late endosomes and lysosomes. The clinical manifestations of the disease vary by age of onset, and severity is often characterized by neurological involvement. To date, no disease-modifying therapy has been approved by the United States Food and Drug Administration (FDA) and treatment is typically supportive. The lack of robust biomarkers contributes to challenges associated with disease monitoring and quantifying treatment response. In recent years, advancements in detection methods have facilitated the identification of biomarkers in plasma and cerebral spinal fluid from patients with NPC, namely calbindin D, neurofilament light chain, 24(S)hydroxycholesterol, cholestane-triol, trihydroxycholanic acid glycinate, amyloid-β, total and phosphorylated tau, and N-palmitoyl-O-phosphocholine-serine. These biomarkers have been used to support several clinical trials as pharmacodynamic endpoints. Despite the significant advancements in laboratory techniques, translation of those advancements has lagged, and it remains unclear which biomarkers correlate with disease severity and progression, or which biomarkers could inform treatment response. In this review, we assess the landscape of biomarkers currently proposed to guide disease monitoring or indicate treatment response in patients with NPC.

Keywords: Niemann Pick Disease Type C, Rare Disease, Biomarkers, Pharmacodynamics, Drug Development

Abbreviations

Aβ, amyloid-β; ALS, amyotrophic lateral sclerosis; AUC, area under the concentration-time curve; calbindin D, calbindin D-28K; CNS, central nervous system; c-triol, cholestane 3β, 5α, 6β-triol; CSF, cerebral spinal fluid; CYP, cytochrome P450; HPβCD, 2-hydroxypropyl-β-cyclodextrin; NfL, neurofilament light chain; NPC, Niemann-Pick disease Type C; NPC-NSS, NPC Neurological Severity Score; OR, odds ratio; TCG, trihydroxycholanic acid glycinate; PPCS, N-palmitoyl-O-phosphocholine-serine; TID, three times daily; SPC, sphongosylphosphorylcholine;

1. Disease Background

Niemann-Pick disease Type C (NPC) is a genetic, rare, highly heterogeneous, and progressive lysosomal disorder characterized by the dysfunction of NPC1 or NPC2 proteins, which leads to the accumulation of multiple lipid species[1, 2]. The broad clinical spectrum ranges from a rapidly fatal antenatal condition to an adult-onset, chronic, neurodegenerative disease. NPC is estimated to occur at a rate of 1 in 120,000 live births[2]. The severity of clinical presentation, primary manifestation, and rate of progression depend on the age of onset and the extent of neurological involvement (e.g., loss of swallowing, speech, and motor function)[3, 4]. The clinical presentation can be categorized by onset into: perinatal (onset < 3 months), visceral-neurodegenerative (early-infantile, onset 3 months to < 2 years), neurodegenerative (late-infantile, onset 2 to < 6 years old), juvenile (onset at 6-15 years old), or psychiatric-neurodegenerative (late-onset, > 15 years old)[5, 6]. Generally, individuals with infantile-onset neurological symptoms have a more aggressive disease course than individuals with juvenile or late-onset disease.

NPC is a cholesterol lipidosis, irrespective of whether it is caused by a genetic variant in *NPC1* or *NPC2*[2]. Approximately 95% of patients with NPC have variations in *NPC1* and the majority (~80%) are missense, resulting in a misfolded or prematurely degraded protein[7-9]. In comparison, other less common (~20%) variations such as frameshift, splicing, and premature stop variants collectively result in a truncated or deficient NPC1 protein and are associated with a severe disease course[5, 10]. In healthy cells, cholesterol is trafficked to the lysosome as low-density lipoprotein cholesterol, hydrolyzed to an unesterified species, and then transported outside the lysosome. However, variants of the *NPC1* or *NPC2* gene lead to impaired processing and trafficking and result in accumulation of cholesterol and other lipids in extra-neural and neural tissues[11]. While glycolipids are the major accumulating lipids in the brains of patients with NPC, it is unclear how NPC1 and NPC2 cooperate to transport cholesterol within the brain and the precise mechanism underlying the manifestation of NPC is not yet fully understood.

Currently, no therapies have been approved in the United States (US) for treatment of NPC. Management focuses on supportive and palliative treatment with multidisciplinary care. Miglustat is an iminosugar that inhibits glucosylceramide synthase which is responsible for the production of sphingolipids. Miglustat is approved by the US Food and Drug Administration (FDA) for Gaucher disease Type 1. Miglustat has been approved for the treatment of NPC in countries outside the US[12]. However, in 2010, the company received a complete response letter from the FDA for miglustat for the indication of NPC[13, 14]. Currently, several therapeutic options are under investigation[15-19].

Considering the varied clinical manifestations and lengthy trials needed to detect changes in clinical outcomes, endpoint selection is often a challenge in trial design. Clinical outcome assessment can be challenging in a nonlinear heterogeneous condition. No clinical outcome measurement has been established as a valid and reliable clinical trial endpoint in NPC. However, the NPC disease severity score is a widely used tool in clinical practice to assess disease severity by calculating a composite score based on neurological impairment measured by domains such as cognitive, speech, memory, fine motor, and swallowing[5]. Currently, no biomarkers are used to inform disease severity or progression in clinical care. Identification and validation of biomarkers that can be used to assess treatment response for drug development and to guide patient care is a critical need for NPC.

2. Current Pharmacodynamic Biomarkers to Inform Treatment Response

Biomarkers may inform diagnosis, prognosis, response to a treatment or intervention, and other aspects of drug response, which make them valuable tools in both patient care and drug development settings. Due to the importance of early intervention to prevent further neurological involvement, increasing attention is being paid to identifying and validating biomarkers that may serve as indicators of response to an exposure or intervention during drug development for NPC, particularly biomarkers that reflect changes in neurodegeneration[20]. In drug development, biomarkers may be used to identify pathological processes amenable to treatment, as pharmacodynamic measurements, or as a surrogate for clinical response, possibly filling a critical need in the development and evaluation of therapies for NPC. Using

techniques such as chromatography and mass spectrometry, many biomarkers have been identified and evaluated for different contexts of use within NPC. Despite advancements in detection methods, the validation of pharmacodynamic biomarkers for NPC has been hampered by the heterogeneous clinical presentation, variability in progression, lack of predictive clinical measurements, and unclear pathophysiology, particularly regarding neurological involvement.

This review summarizes the available literature on biomarkers of NPC that may have the potential to inform treatment response and monitor or assess disease severity. While the majority of data are focused on biomarkers in patients with NPC1, it is unlikely that these biomarkers would differ in patients with NPC2 due to the lack of biomarker specificity and similar pathophysiology. We focused our search to biomarkers associated with neurodegeneration in patients with NPC using publicly available information (Figure 1). We subsequently prioritized biomarkers used in clinical trials for NPC based on their relationship to disease pathology and those that may be useful assessments of disease progression and clinical outcomes (Table 1). For each biomarker, we provide relevant background, describe results from studies (including trials in humans and animal models), and conclude with an overall assessment to highlight potential areas of future research.

2.1. Calbindin D

A hallmark characteristic of NPC is the intracellular accumulation of cholesterol, however, the exact mechanism responsible for cerebellar Purkinje cell death and cerebellar dysfunction remains unclear[21]. Calcium-binding protein calbindin D-28K (calbindin D), a major calcium-binding protein that acts as a cytosolic calcium buffer, is present in high concentrations in the cerebellum. Reductions in calbindin D immunoreactivity have been observed in cerebral spinal fluid (CSF) of spinocerebellar ataxia-1 transgenic mice prior to the loss of neuronal cells and the onset of ataxia, potentially indicating nonspecific neurological disease progression[22, 23]. Decreased calbindin D immunoreactivity in cerebellar Purkinje cells and cell loss have also been observed in feline and murine NPC models[24]. Additionally, an increase in the CSF concentration of calbindin D has been described as a marker of neurological diseases

such as NPC, particularly those involving the cerebellum[25-27]. Given its high expression in cerebellar Purkinje neurons and association with cerebellar ataxia, a notable symptom of NPC, calbindin D is a biomarker of particular interest[27, 28].

CSF calbindin D was evaluated as a potential biomarker for disease progression and treatment response to HPβCD in pre- and post-symptomatic conditions in a feline model[29]. CSF calbindin D concentrations of untreated NPC1 cats were significantly higher than normal age-matched cats. Elevated concentrations were found prior to neurological manifestations at 3-weeks of age and were 10-fold higher at end-stage disease compared to normal cats[29]. After intracisternal treatment with 120 mg of HPβCD (4000 mg/kg brain weight) administered every other week in NPC1 cats, the calbindin D concentration was reduced to a concentration similar to normal cats. The reduction of calbindin D concentration was delayed in cats that received HPβCD after symptoms were present compared to cats treated pre-symptomatically. This study further compared CSF calbindin concentration in patients with NPC1 to controls. In patients with NPC1, CSF calbindin D concentrations were significantly elevated ($p<0.0001$, 4.78 vs 0.76 ng/mL) compared to healthy controls. Paired samples of pre- and post-miglustat therapy (200 mg TID adjusted by body surface area) for the subset of patients with CSF samples available showed a significant reduction in calbindin D concentrations ($p=0.011$), despite no significant difference in calbindin D concentration when comparing untreated NPC patients to miglustat-treated patients[29]. This difference in treatment effect observed within subjects and across subjects may be attributable to clinical severity.

Campbell et al investigated the CSF proteome to identify potential biomarkers for NPC. Among the proteins whose expression was altered in the CSF of patients with NPC compared to controls, the concentration of calbindin D was 1.7-fold higher in non-miglustat treated patients (adj $p=0.0162$). In the combined group of patients with NPC, regardless of treatment status, calbindin D concentration was elevated by 1.6-fold compared to controls (adjusted $p=0.000052$)[27]. Although calbindin D was not one of the top 50 proteins with increased expression, the expression of calretinin and secretagogin, which are two other proteins in the hexa-EF-hand family, was 3.4-fold and 2-fold higher in patients with NPC,

respectively. Taken together, calbindin D, calretinin, and secretagogin appear to have increased CSF concentrations in patients with NPC[27].

Calbindin D was evaluated in a phase 1/2a clinical trial[19]. This non-randomized open-label dose escalation study investigated the safety, pharmacodynamics, and efficacy of intrathecal HPβCD (ranging from 50-1200 mg) administered every other week or monthly in patients with NPC aged 2-25 years. Patients with NPC had significantly higher CSF calbindin concentrations at baseline compared to healthy controls values derived from literature (532 ng/mL vs 0.76 ng/mL). Calbindin D concentrations at the last treatment (13 or 18 doses) compared to baseline showed a decrease in nine subjects, increase in one subject, and no difference in four patients; collectively, a significant decrease in concentrations (-385 ng/mL, p=0.004) was found[19].

Although few studies have evaluated calbindin D as a biomarker for NPC, studies have demonstrated a consistent directionality of increased CSF concentrations in patients with NPC compared to a control group and trends towards decreased concentrations with treatment of two different drugs with two different mechanisms of action. Findings of increased concentrations in NPC align with the understanding of disease progression, specifically the notable cerebellar Purkinje cell death; therefore, calbindin D may represent a potential pharmacodynamic biomarker. However, mechanistic underpinnings of the observed delay in calbindin D reduction in symptomatic felines and differing trajectories following treatment remain uncertain. It is also unclear how CSF calbindin D concentrations correlate with the NPC disease severity score and other clinical scales used in clinic.

2.2. Neurofilament light chain

Neurofilaments are cytoskeletal proteins composed of four subunits, a heavy (190-210 kDa), medium (150 kDa), light (68 kDa), and α-internexin. Neurofilament light chain (NfL) is the most abundant and soluble subunit that can be quantified[30]. Neurofilaments are involved in axonal radial growth and larger myelinated axons express more neurofilaments[31]. NfL and other neurofilaments are likely released into

the CSF during neuroaxonal damage in an age-dependent manner, as they play a role in axonal stability, radial growth, and communication with other proteins and cellular organelles[32, 33]. In neurodegenerative diseases such as amyotrophic lateral sclerosis (ALS) and Alzheimer disease, NfL has been assessed in plasma and CSF as an indicator of disease progression and the conversion from pre-symptomatic to symptomatic stages[34-39]. The degree of damage is speculated to be proportional to the NfL concentration released into the blood and CSF, suggesting that NfL is a prognostic and response biomarker for certain neurological diseases[40].

Although NfL concentrations are approximately 50-fold lower in plasma than in CSF, elevations in both CSF and plasma NfL concentrations have been observed in patients with NPC[40-42]. Cross-sectional and longitudinal CSF samples from 116 patients with NPC and age-matched non-NPC controls showed that the median baseline CSF NfL concentration was 6.9-fold higher in patients compared to controls (1152 [680-1840] vs 167 [82-372] pg/mL)[43].

Using pooled results from a National Institute of Health (NIH) natural history study, a Phase 1/2 and 2/3 clinical trial for intrathecal HPβCD, and an expanded access program, Agrawal et al. reported that patients with NPC had substantially elevated CSF NfL concentrations compared with non-NPC controls of similar ages[43]. Interestingly, between patients with and without neurological involvement, no differences in CSF NfL concentrations were detected. However, the group of samples from patients without neurological symptoms was small (n=6). Baseline CSF NfL concentrations were significantly correlated with the severity of NPC presentation, as characterized by a 17-domain (r_s= 0.33, 95% CI= 0.16-0.49) and 5-domain NPC Neurological Severity Score (NPC-NSS) (r_s=0.34, 95% CI= 0.17-0.49)[43]. Odds ratios (ORs) were calculated as the risk of more severe disease associated with elevated NfL concentrations. Higher CSF NfL concentrations were associated with increased disease severity across several outcomes including the 17- and 5-domain NPC-NSS, though not the cognitive domain of the latter when this domain was analyzed separately. For patients who received HPβCD, there were no changes in CSF NfL concentrations at the last visit (OR= 1.25, 95% CI= 0.99-1.58) and CSF NfL

concentrations were found to increase over time after adjusting for miglustat use (OR= 1.35, 95% CI= 1.09-1.67)[43]. For patients who received miglustat, a reduction in CSF NfL concentrations (OR=0.77, 95% CI= 0.62-0.96) was observed over the follow-up period (up to 13.5 years)[43].

During investigations of plasma NfL concentrations in 75 healthy volunteers compared to 26 patients with NPC, patients with NPC had significantly elevated plasma NfL concentrations compared to controls even after patients were age-matched by < 18 years of age and ≥ 18 years of age[44]. Ten of the 24 adults and nine of the 15 pediatric samples obtained from patients with NPC were asymptomatic at the time of sampling and, consequently, both groups were further stratified by the presence and absence of neurological involvement[44]. It was shown that NfL concentrations in plasma were significantly elevated in patients with NPC with neurological involvement compared to those without neurological involvement in both pediatric and adult groups. For adult patients with NPC without neurological involvement compared to age-matched healthy volunteers, plasma NfL concentrations were similar. For pediatric patients with NPC without neurological symptoms compared to age-matched healthy volunteers, NfL concentrations were similar between groups in all but two patients. Despite the small cohort of patients in this study, these data suggest that plasma NfL concentrations may be a potential biomarker to detect severity of disease particularly for those with neurological involvement.

Data on NfL as a treatment response biomarker is limited, although recent work has shown a potential correlation between NfL concentrations and disease severity scores. Despite this effort, current limitations of NfL as a response biomarker include the need to control for NfL concentration with increasing age, unclear relationships with disease pathology and neurological outcomes, the need for replicated studies that correlate outcomes with biomarker changes, and limited data demonstrating an association between CSF and plasma NfL concentrations. It is unclear to what degree plasma elevation reflects peripheral versus central nervous system (CNS) disease and whether incremental differences in CSF or plasma concentrations can inform prognosis or treatment response. A key challenge in obtaining adequate data to

support the use of NfL as a biomarker to inform treatment response is the generalizability of results from healthy adult studies to children due to the effect of age on NfL concentrations.

2.3. 24(S)-hydroxycholesterol

Lysosomal cholesterol accumulation is a major characteristic of NPC pathophysiology, however, there is limited information on whether a change in sterol flux across the blood-brain barrier might contribute to neurodegeneration. 24(S)-hydroxycholesterol is a dominant oxysterol that maintains cholesterol homeostasis. Cytochrome P450 46A1 (CYP46A1) converts cholesterol to 24(S)hydroxycholesterol, is predominantly expressed in neurons, and is responsible for approximately 40% of brain cholesterol metabolism[45, 46]. Brain concentrations of 24(S)-hydroxycholesterol directly correlate with systemic cholesterol concentrations, and the concentration gradient is a major driver of 24(S)-hydroxycholesterol diffusion across the blood-brain barrier[47, 48]. It is speculated that 24(S)-hydroxycholesterol may favor membrane cholesterol accessibility and therefore indirectly alter the membrane structure and increase the extent of cholesterol transport within the endoplasmic reticulum membrane[49]. Additionally, 24(S)-hydroxycholesterol is a ligand of nuclear receptors liver X receptor (LXR) α and β, which suppresses brain cholesterol biosynthesis. In turn, this reduces the synthesis of cholesterol mediated by LXR activation and enhances the expression and synthesis of cholesterol facilitators and transporters, ApoE and ABCA1/ABCG1, in astrocytes[50-53].

An analysis of plasma from ten patients with NPC enrolled in a natural history study demonstrated that 24(S)-hydroxycholesterol is significantly lower compared to age-matched healthy controls[54]. Plasma 24(S)-hydroxycholesterol concentrations were similar to other lysosomal storage diseases such as GM1 and GM2 gangliosidosis, infantile neuronal ceroid lipofuscinosis, and Gaucher disease, consistent with the cortical involvement of these neurodegenerative diseases. Notably, 24(S)-hydroxycholesterol could not be detected in the CSF of NPC or control subjects, potentially reflecting the reduced 24(S)-hydroxycholesterol synthesis or a limitation in detection techniques[54]. While significantly lower mean plasma concentrations of 24(S)-hydroxycholesterol were found (97.7 [34.8-234.1] ng/mL in controls vs

75.1 [19.3-209.3] ng/mL in patients with NPC), considerable overlap in concentrations demonstrates that 24(S)-hydroxycholesterol may not be sufficient to distinguish unaffected individuals from patients with NPC.

In a study using 9-week *Npc1* knockout mice, 24(S)-hydroxycholesterol concentrations were 35% lower in whole brain tissue and 2.8-fold higher in the liver compared to control mice[54], demonstrating that elevated steady-state levels are potential manifestations of hepatocellular injury or increased clearance of plasma oxysterols[54, 55]. Furthermore, *Npc1* $^{-/-}$ mice stereotactically administered 6 mg/kg HPβCD into the left lateral ventricle demonstrated a dose dependent increase in plasma 24(S)-hydroxycholesterol concentrations compared to control mice administered artificial CSF over 7 days[56]. This change was associated with a significant reduction in the cholesteryl ester content in the brains of *Npc1* $^{-/-}$ mice. In 4- to 5-week-old *Npc1* $^{-/-}$ mice treated with 6 mg/kg of HPβCD intracerebroventricularly every other week (2 doses), significant elevations in brain and plasma 24(S)-hydroxycholesterol concentrations were found. 24(S)-hydroxycholesterol concentrations returned to baseline within 17 days, however, lysosomal cholesterol in the brain did not reaccumulate during that period[56]. Similar elevations in plasma and CSF 24(S)-hydroxycholesterol concentrations were demonstrated during intrathecal administration of HPβCD in 2-week-old NPC1 cats treated every other week until 11 weeks of age with increasing concentrations of HPβCD (3, 30, or 120 mg)[56].

In a case study of a single 12-year-old patient, plasma and CSF 24(S)-hydroxycholesterol concentrations were increased and improvements in hearing and vertical gaze were observed after the first 5 intrathecal administrations of 200 mg HPβCD every other week[57]. Subsequently, a Phase 1 study evaluating 50 mg of HPβCD administered intracerebroventricularly to patients with NPC1 found that the maximum concentration of CSF 24(S)-hydroxycholesterol was 21- to 50-fold higher than baseline at 0.25-0.5 hours post-dose and the area under the concentration-time curve (AUC) increased from 0-72 hours post-dose from 1.6- to 2.9-fold compared to saline[58]. Plasma concentrations of 24(S)-hydroxycholesterol also

increased from 1.43- to 1.75-fold baseline values at 3-8 hours post-administration of HPβCD and the AUC increased 6-16% in plasma compared to saline.

In an 18-month, open-label Phase 1/2a study of escalating (50-1200 mg) intrathecal HPβCD doses administered to 14 patients with NPC, plasma and CSF 24(S)-hydroxycholesterol were the primary pharmacodynamic markers measured pre-dose, at 8-, 24-, 30-, 48-, and 72-hours post-dose[19]. The plasma 24(S)-hydroxycholesterol concentrations were variable, with a majority of participants having concentrations above those of individuals who received the saline control. Despite the variability, higher plasma 24(S)-hydroxycholesterol concentrations were detected at higher doses and a significant dose dependent effect was observed at 900 mg and 1200 mg. CSF 24(S)-hydroxycholesterol demonstrated robust elevation and three participants had a > 2-fold increase in concentrations 72-hours after the administration of 600-900 mg of HPβCD[19].

The dominant oxysterol in the brain is 24(S)-hydroxycholesterol, which is an attractive marker for neuronal function and cholesterol accessibility by favoring cholesterol efflux[49]. The majority of 24(S)-hydroxycholesterol produced in the brain enters systemic circulation and is delivered to the liver for hepatic metabolism, thereby enabling 24(S)-hydroxycholesterol to be indicative of cholesterol homeostasis in the brain. However, 24(S)-hydroxycholesterol concentrations are subject to significant variability based on age, comorbidities, leakage of the blood-brain barrier, or differences in metabolism, potentially undermining the use of 24(S)-hydroxycholesterol as a pharmacodynamic biomarker. Additionally, increased plasma and/or CSF 24(S)-hydroxycholesterol concentrations in response to treatment with HPβCD have been observed across murine models, feline models, and in humans. Data demonstrating changes in 24(S)-hydroxycholesterol concentration are not available for other potential therapies and the relationship with disease severity scores or clinical assessment has not been investigated.

2.4. Cholestane-triol

An excess of intracellular cholesterol along with heightened oxidative stress in NPC promotes the nonenzymatic formation of oxysterols[54]. An oxysterol, cholestane-3β, 5a, 6β-triol (c-triol), has largely been investigated as a potential biomarker for non-invasive screening and diagnosis of NPC[59]. Reported screening strategies with c-triol include a combination approach with other biomarkers and methodologies[5, 60], such as the previous use of chitotriosidase activity[61], filipin staining[61, 62], 7-ketocholesterol, and a composite of c-triol, trihydroxycholanic acid glycinate (TCG), and N-palmitoyl-O-phosphocohlineserine (PPCS)[61-64] These screening strategies have been applied in infants, adolescents, and adults with upper bounds of the c-triol reference range varying from 32 to 60 ng/mL across studies[61, 62, 65, 66].

Studies have demonstrated elevated c-triol concentrations in patients with NPC[54, 62]. Porter et al. studied c-triol concentrations in a murine model, feline model, and in humans[54]. Plasma, liver, and brain c-triol concentrations were elevated in an NPC murine model, with age-dependent increases noted in the cerebellum. In a feline NPC model, serum c-triol concentrations significantly decreased following HPβCD treatment. In ten patients with NPC in an NIH-natural history study, elevated plasma c-triol concentrations were found. In a separate cohort of patients with NPC (n=25) compared to 1:1 age-matched controls, the findings were replicated with a relative 10-fold increase in plasma c-triol concentrations in patients with NPC. Significant elevations in CSF c-triol were also observed in patients with NPC compared to pediatric controls[54]. In addition, an approximate 2-fold increase in plasma c-triol concentrations was reported in NPC1 heterozygotes compared to non-carriers. Plasma c-triol concentration varied by age ($r = -0.40$, $p < 0.05$) and was significantly correlated with disease severity ($r = 0.39$, $p < 0.05$)[54].

Cooper et al. found that patients with NPC who were diagnosed at less than two years of age had higher mean plasma c-triol compared to older patients (449.0 ng/mL [n=9] vs 83.9 ng/mL [n=10])[62]. These results may be reflective of a more severe phenotype that is associated with younger age of onset. To further relate c-triol concentrations to disease severity, Stampfer et al. developed the NPC clinical

database score to quantify disease severity and found a positive correlation between scores and plasma oxysterol concentrations, including c-triol (R^2= 0.274)[3].

Reduction in c-triol concentrations has been investigated as a biomarker in response to therapy. Decreases in plasma and brain c-triol concentrations were observed in *Npc1*$^{-/-}$ mice at 17 days post-HPβCD which suggests that c-triol may be indicative of chronic cellular cholesterol storage[56]. Similarly, in two patients with NPC treated with intravenous HPβCD, plasma triol concentrations significantly decreased by 46% at 25 months and 70% after 24 months of treatment[56]. In a Phase 2/3 trial of pediatric patients with NPC treated with arimoclomol, serum c-triol concentrations trended downward for patients on the treatment versus placebo arm at 12 months (mean [SE] treatment difference of -5.50 [4.46] ng/mL); however, the differences were not statistically significant[16]. Another study reported average plasma c-triol concentrations below the reference value (58.58 ng/mL with reference value of <60 ng/mL) in miglustat-treated patients (n=5); however, concentrations were significantly higher than healthy controls (n=52; p<0.01)[65].

Oxysterols like c-triol are well established for the diagnosis of NPC and remain elevated throughout the course of NPC[54]. These findings are consistent with the cholesterol accumulation and oxidative stress associated with NPC. In contrast to identification of patients with NPC, few studies have investigated c-triol concentrations in relation to treatment response. Although there was a reduction in c-triol concentrations in response to three different therapies, the extent of reduction varied and one of three studies lacked statistical significance, and results remain to be replicated. However, few studies have correlated c-triol concentrations with disease severity. It is unclear how changes in c-triol concentrations during treatment correspond to clinical assessments.

2.5. Trihydroxycholanic acid glycinate

C-triol enters circulation and is hepatically metabolized to a byproduct, trihydroxycholanic acid, and a glycinated derivative, trihydroxycholanic acid glycinate (TCG)[60]. Elevated c-triol concentrations correspond with elevation in TCG which has been found in patients with NPC[64, 67]. The development

of a non-invasive blood diagnostic panel that detects TCG has accelerated diagnosis through dried blood spot detection and newborn screenings[60, 67].

Beyond the detection of disease, plasma TCG has been assessed in samples from clinical trials to assess effects of treatments on concentrations. Plasma TCG concentrations collected from a Phase 1/2 trial and open-label expanded access protocol for every other week of intrathecal HPβCD (200-1200 mg) as well as single patient INDs for intravenous HPβCD (500-2000 mg/kg/week) found that all patients with NPC1 were above a plasma TCG diagnostic threshold of 18.5 ng/mL except for a single patient without neurological involvement[63]. Additionally, plasma TCG concentrations were influenced by sex, as female patients with NPC1 had 31% lower concentrations than male patients. Despite the sex differences in TCG concentrations, patients with NPC1 had an average 15% lower TCG plasma concentrations following intrathecal HPβCD administration at the last study visits[63]. However, plasma TCG concentrations were not significantly correlated with NPC neurological disease severity scores, or annual severity increment scores and therefore, the relationship with clinical benefit is unclear. Miglustat treatment (200 mg TID adjusted for body surface area) did not lead to significant differences in plasma TCG concentrations and concentrations remained above the diagnostic threshold[63].

Plasma TCG concentrations had variable trends in response to treatments, with reductions following HPβCD and no change following miglustat[63]. Although TCG and c-triol have primarily been used in the context of diagnostics, they potentially provide a marker for peripheral tissue involvement[5]. A future challenge is demonstrating that this biomarker, along with other metabolites, reflects the impact on the CNS compared to peripheral tissue given the metabolite concentration is significantly lower in the brain than in the liver[60].

2.6. Amyloid-β

Amyloid-β (Aβ) is a small protein (37-49 amino acids) that is cleaved from a β-amyloid precursor protein by β- and γ-secretases and is widely associated with a pathophysiological role in Alzheimer disease[68, 69]. Different Aβ isoforms (e.g., Aβ38, Aβ40, Aβ42) are denoted based on N- and C-terminal truncation

by a variety of secretases, where Aβ38, Aβ40, and Aβ42 are the most prominently known C-terminally truncated peptides in the CSF with varying peptide length and solubility[70]. Peptide solubility has been associated with disease severity in neurodegenerative diseases, such as Alzheimer disease[71, 72]. Accumulating evidence suggests that there is an association between Aβ and cholesterol homeostasis. For instance, increased dietary cholesterol led to a decrease in secretion of Aβ in various neuroblastoma cells and primary neuronal cells. Since the late 1990s, there has been a growing body of evidence demonstrating the association between Aβ and cholesterol metabolism, reflected in both NPC and Alzheimer disease. A positive correlation has been shown between Aβ concentrations and cholesterol concentrations in vitro and in vivo[73, 74]. Additionally, Aβ directly interacts with cholesterol in vitro, but the interaction sites have not been identified[75]. Consistent with in vitro models, the brains of *Npc* -/- mice exhibit Aβ aggregation, particularly in late endosomes[76]. While a bidirectional link has been speculated, the mechanism underlying this interaction remains unclear.

In patients with NPC, it has been shown that Aβ concentrations in CSF are significantly elevated, shifting towards the Aβ42 isoform, an insoluble peptide, compared to individuals without NPC[77]. While Aβ42 was the most elevated isoform, other isoforms such as Aβ38 and Aβ40 were elevated compared to the control samples. However, several patients with NPC had concentrations of Aβ that overlapped with the control individuals. Mattsson et al. assigned scores according to disease severity demonstrating that individuals with high disease burden had higher Aβ concentrations. Patients with NPC treated with miglustat had lower Aβ42 concentrations compared to untreated patients with NPC. However, all other isoforms of Aβ remained consistent, despite miglustat treatment[77].

In contrast, analysis of the brains of patients with NPC indicated that other forms of amyloid protein precursors accumulate in cerebellar Purkinje cells and early endosomes, while Aβ42 does not[78]. In late endosomes and hippocampal pyramidal neurons, Aβ42 was the major accumulating species of amyloid. Thus, the location of abnormal lipid trafficking in NPC may contribute to the presentation.

Using a congenital NPC model in cats compared to human CSF samples, Mattsson et al. evaluated the altered distribution of CSF Aβ deposition in NPC, findings of which may highlight species differences[79]. The authors noted the relative concentrations of Aβ37, Aβ38, and Aβ39 trended lower in NPC cats compared to controls. In NPC cats treated with HPβCD, CSF Aβ concentrations decreased over time including Aβ42, Aβ38, and Aβ40, while Aβ16 increased compared to untreated NPC cats[79]. However, interpretation of these findings is difficult given the small sample size (n=3 NPC cats) and overlapping concentrations. In twin patients with NPC, CSF samples were evaluated to determine the Aβ concentrations before and after HPβCD and miglustat co-treatment. The patients initially received 100 mg/kg HPβCD intravenously and the dose was titrated up to 2,500 mg/kg. Patients also received intrathecal HPβCD (175 mg or 375 mg) administered in addition to the intravenous dosing every 2 weeks. The CSF concentrations of isoforms Aβ1-42, Aβ38, Aβ40, Aβ42, and amyloid precursor protein decreased in a time-dependent manner for both patients despite the pattern of distribution remaining unchanged.

CSF Aβ concentrations, specifically Aβ42, may decrease in response to treatment, but the magnitude of that reduction and meaningfulness related to clinical assessment is uncertain. The underlying mechanism of action associated with the relationship between NPC and Aβ deposition remains unclear, reducing its utility as a pharmacodynamic biomarker. Furthermore, it remains unclear whether the association is related to cholesterol efflux or synthesis, and thus conclusions are difficult to draw. Despite the fact that Aβ processing appears altered in NPC, dense core Aβ plaques are not observed in individuals with NPC[80]. The location and trafficking of lipids and location of deposition of Aβ may contribute to the phenotype. More investigations are necessary to uncover the unique degradation pathway of Aβ in patients with NPC.

2.7. Total and Phosphorylated Tau

Loss of NPC1 and NPC2 function modifies the neuronal storage of lipids and alters morphology, preceding axonal and neuronal damage[21]. Purkinje cells in the cerebellum, basal ganglia, and thalamus

are often the first affected and are the most susceptible neurons to NPC-induced lipid accumulation and neuronal injury[81, 82]. Thus, to further investigate biomarkers for neurodegeneration that may be potentially useful for clinical trials, CSF concentration of total tau has been suggested as a marker for neurodegeneration while phosphorylated tau may be specific as a marker for neurofibrillary tangles, correlated with cognitive decline[83, 84].

The relationship between NPC and total tau has been investigated using tau-imaging. Multiple patients with NPC have shown elevated tau burden with the distribution of tau varying between the mesial temporal, temporoparietal, and rest of the neocortex[85]. A significant correlation was demonstrated between tau burden in the mesial temporal region and age, age of onset, and between tau burden in the rest of the neocortex and duration of symptoms. However, not all patients with NPC demonstrated an increased tau burden despite severe cognitive impairment and long duration of neurological symptoms, potentially an artifact of age.

In a case study of a 5-year-old patient and an 11-year-old patient with advanced stage and moderately advanced stage NPC, respectively, CSF biomarkers such as total tau and phosphorylated tau, NfL, and 24(S)-hydroxycholesterol were assessed[86]. Baseline total tau and phosphorylated tau were all substantially elevated. Both patients were treated with miglustat orally and HPβCD intravenously and intrathecally. Improvements in visceral disease were associated with a decline in total tau and phosphorylated tau, particularly in the 11-year-old patient[86]. Other biomarkers investigated remained unchanged.

In an observational study of 16 patients with NPC treated with and without miglustat (five patients untreated at baseline and treated at follow-up, six patients treated at baseline and at follow-up, and five patients untreated at baseline and follow-up), CSF concentrations of total tau and phosphorylated tau were evaluated[87]. The untreated group had later onset of neurological symptoms, older age at CSF collection, longer disease duration, and higher baseline disease score compared to the other two groups. Patients untreated at baseline had the highest baseline CSF total tau concentrations compared to other groups.

There was a significant reduction in CSF total tau concentrations at follow-up for patients treated with miglustat therapy[87]. Patients with NPC on prior miglustat therapy and continuing treatment had the lowest baseline CSF total tau at the collection time compared to patients without miglustat treatment. The decrease in total tau was associated with initiation of miglustat therapy, as demonstrated by stable total tau concentrations in the untreated patients[87]. However, out of all 16 patients, there was no significant difference in total tau concentrations between untreated and treated patients at the time of follow-up, potentially demonstrating that miglustat does not fully prevent neurodegeneration or disease progression. Phosphorylated tau did not differ significantly over time or between treated and untreated patients[87].

A phase 1, randomized, double-blind, parallel-arm 14-week study enrolled adult patients with confirmed NPC1 and evidence of systemic involvement of disease who received 1500 mg/kg or 2500 mg/kg of HPβCD administered intravenously every 2 weeks[88]. Plasma and CSF sampling were performed after the first and seventh infusion to assess biomarkers. In 60% of patients who received 7 doses of either 1500 mg/kg or 2500 mg/kg (6/10), there was a post-treatment reduction in CSF total tau with the mean concentration increasing after the first infusion and decreasing after the 7th infusion. Eighty-two percent (9/11) of patients who had available data demonstrated an increase in CSF total tau after the first infusion. Hastings et al. suggested that the increase in total tau after the first infusion may be due to the release of a tau bolus from neurons with a downward trend in total tau after the last infusion suggesting the potential for HPβCD to reduce the rate of degeneration in neurons[88]. Total tau concentrations did not show a consistent decline in all subjects following the last infusion and rapid changes in the concentrations may make capturing the impact challenging.

Overall, CSF total tau has been shown to be a potential biomarker for neuronal apoptosis, degeneration, and loss[89]. Despite its role in neurological diseases such as Alzheimer disease, the variable response among individuals with NPC represents unique challenges with interpreting its use as a biomarker.

2.8. N-palmitoyl-O-phosphocholine-serine

Screening and diagnosis of NPC incorporate a novel lipid known as N-palmitoyl-O-phosphocholine-serine (PPCS) [5, 60, 90]. Initially coined as lysosphingomyelin-509, upon further structural analysis PPCS was elucidated as the most likely and abundant structure. Throughout this manuscript, we will refer to PPCS instead of lysosphingomyelin-509 to reflect the most accurate lipid species. Elevations in PPCS plasma concentrations in patients with NPC have been observed, revealing a potentially undiscovered metabolic pathway[92, 93]. PPCS has emerged as a novel biomarker that is utilized first-line for NPC screening (along with other various lysosphingolipids in a multiplex assay) and has potential to assess treatment response in NPC[5].

In a Phase 1, double-blind, parallel group study, patients with NPC received 1500 mg/kg or 2500 mg/kg of intravenous HPβCD every 2 weeks for a total of 14 weeks (7 total doses)[88]. Pharmacodynamic markers, including plasma PPCS, were assessed at Baseline, and Week 2, 4, 8, 12, and 14. Findings suggest that subjects had a significant reduction in plasma PPCS concentrations, with a 46.8% reduction at Week 12, which was not dose dependent[88].

In a phase 2/3, 12-month, randomized, double-blind, placebo-controlled trial of patients with NPC aged 2 to 18 years, patients were randomized to receive arimoclomol in a 2:1 ratio[16]. At baseline, all subjects had elevated plasma PPCS concentrations compared to healthy volunteers (666 ng/mL vs 10 pg/mL), which was inversely correlated with age of neurological onset and associated with serum c-triol at baseline and differences at Month 12. Patients treated with arimoclomol had significant reduction in plasma PPCS concentration compared to those receiving placebo at Months 6 and 12.

In addition to its use as a diagnostic marker, PPCS holds promise to inform and guide treatment response[94]. Elevated concentrations in patients with NPC at baseline were decreased in response to miglustat and HPβCD, as described. Research is warranted to untangle the relationship between PPCS elevation and disease severity and provide evidence that it may be used to measure disease progression and treatment response.

3. Discussion

Drug development for NPC is challenging due to the heterogeneous presentation, nonspecific early symptoms, progressive nature, complex pathophysiology, and unclear relationship between biomarkers and diagnosis, prognosis, and clinical benefits of drug treatment. A variety of biomarkers have potential as pharmacodynamic biomarkers to inform treatment response and monitor or assess disease severity such as calbindin D, 24(S)-hydroxycholesterol, c-triol, TCG, NfL, PPCS, amyloids, total and phosphorylated tau, which have been reported throughout this review (Figure 1). Additional novel biomarkers have been recently identified, such as ubiquitin C-terminal hydrolase-L1[95]. In this review, we outlined the available nonclinical and clinical data supporting the potential use of candidate biomarkers for drug development and treatment monitoring purposes. While several promising biomarkers have been identified, additional data are necessary to support their routine use.

In the context of drug development, disease-related or treatment-related biomarkers and the therapeutic mechanisms of action should share the same biological pathway, enabling insight into the drug mechanism of action and potential impact on disease outcomes [96]. NPC pathophysiology is not fully understood, and the biomarkers discussed within this review are not specific to NPC; thus, there are challenges with understanding their role and impact on this disease.

Given that the clinical course of NPC is a result of aberrant cholesterol trafficking, cholesterol-associated biomarkers (e.g., 24(S)-hydroxycholesterol) appear closely linked to NPC pathophysiology, which strengthens their potential as pharmacodynamic biomarkers. 24(S)-hydroxycholesterol maintains cholesterol homeostasis in the brain, while c-triol and TCG are derivatives of excess cholesterol and oxidative stress in the liver. Overall, c-triol and TCG are several steps removed from cholesterol accumulation and induce changes in autophagy-lysosome function which potentially contribute to neurodegeneration in NPC[97]. These biomarkers are not specific to NPC and have shown baseline elevation in other diseases such as pediatric onset of cholestasis, other biliary atresia, and acid sphingomyelinase deficiency[64, 99, 100]. Despite the lack of specificity, they are accessible and can be

readily measured, and in combination with other biomarkers may shed light on a drug's clinical pharmacology and be useful indicators of treatment effects. Elucidation of their potential use as pharmacodynamic biomarkers would benefit from evaluation in natural history studies to understand their relationship with clinical outcomes.

Preliminary evidence suggests that protein biomarkers may have a role in monitoring CNS treatment effectiveness compared to metabolic biomarkers despite being potentially reflective of neurodegeneration and not specific to NPC. Examples discussed include NfL and calbindin D, both of which were found to be elevated at baseline and decreased in response to treatment in patients with NPC[19, 29, 42, 43]. While CSF is ideal for measuring CNS involvement, it is unclear how plasma concentrations of these biomarkers reflect peripheral versus CNS involvement. Additionally, plasma concentrations are not always reflective of CSF concentrations and more data is warranted demonstrating a direct association between CSF and plasma concentrations.

A recent study revealed that there may be a correlation between ventricular volume and CSF protein production, thus potentially confounding the observed relationships and limiting the use of many of these biomarkers [101]. For instance, frontal lobe atrophy has been noted in some patients with NPC[102]. This is consistent with the findings of Ott et al. for 288 subjects, who showed a negative association between ventricular volume and CSF amyloid proteins and tau in controls and patients with Alzheimer disease[103]. Along with confounding neurodegenerative variables and age-dependent elevations, plasma and CSF proteins can be distinct, and their expression may differ by an order of magnitude between plasma compared to CSF.

Collection of CSF protein via lumbar puncture has ethical and feasibility considerations, particularly for pediatric populations. Albert et al. found that, out of 59 patients with NPC treated with 2935 infusions, 55.9% of patients had no adverse events following a lumbar puncture, 44.1% had an adverse effect reported at some point, and adverse events occurred after 3.3% of lumbar puncture procedures[104]. Although lumbar punctures are a drug delivery method used in the development of drugs for NPC and are

frequently used to obtain CSF, mild complications are possible in both adult and pediatric populations with additional considerations and complications possibly due to sedation[105]. Postdural puncture headaches are often the most commonly reported adverse event reported after lumbar puncture procedures[106].

Further efforts to understand the biomarkers' relation to the natural history of the disease supported by a mechanistic understanding of how the biomarker is involved in the disease pathway is necessary to accelerate advancements in treatments. The adequacy of the assay in terms of its analytical performance may impact the ability to discern treatment response leading to inaccurate conclusions. An overarching challenge yet-to-be-addressed is whether these biomarkers are clinically sensitive enough to detect a treatment response, or differentiate effects of different drug dosages. Additionally, without a clear relationship with clinical outcomes, even if such biomarker measures are objective, it is important to evaluate drug effectiveness in active comparator in studies. Furthermore, the ability to investigate these biomarkers and anchor them to clinical outcomes is challenged by the lack of validated clinical assessment tools.

A pharmacodynamic biomarker may be specific to a drug or drug class, particularly if it is proximal to the drug target. In contrast, a pharmacodynamic biomarker that is further downstream from the drug target may not be sensitive to the drug effect or change in dose. It is our opinion that more comprehensive panels of biomarkers, rather than a single biomarker for all contexts of use and all drugs under investigation, are needed to robustly characterize patient response and optimize the treatment regimen. Combining biomarkers that reflect pathophysiological cholesterol trafficking and oxidative stress with lipid biomarkers may provide insight for drug development. For instance, results from a Phase 1/2a study of escalating (50-1200 mg) intrathecal HPβCD doses administered to patients with NPC demonstrate a treatment effect depicted by reduction in CSF calbindin D and increases in CSF and plasma 24(S)-hydroxycholesterol, potentially strengthening the support of a treatment effect[19]. Additional considerations which may provide a path forward to bridge the gap in knowledge about the impact of

these biomarkers in the disease pathophysiology and utility of these biomarkers to assess drug activity, include 1) collection and banking of specimens in natural history studies to measure relevant biomarkers using validated assays, 2) combining data from available clinical studies to reduce noise and better understand natural changes in these biomarkers during the course of disease, and 3) consistent evaluation of promising PD biomarkers in trials of therapeutic interventions [107].

Acknowledgements

The authors thank the scientists who have made contributions to the field but have not been cited because of space limitations.

Funding

No funding to declare.

Author information

Sydney Stern and Karryn Crisamore have contributed equally to this work.

Authors Contributions

SS, KC, RS, and MP wrote or contributed to the writing of the manuscript. All authors approved the final manuscript.

Disclaimer

This article reflects the views of the authors and should not be construed to represent FDA's views or policies.

Ethics Declaration:

Ethics approval and consent to participants

Not applicable.

Consent for publication

Not applicable

Availability of data and materials

Not applicable.

Competing interests

All authors declare no competing interests for the article.

References

1. Lloyd-Evans E, Platt FM. Lipids on trial: the search for the offending metabolite in Niemann-Pick type C disease. *Traffic* 2010, 11(4):419-428.
2. Vanier MT. Niemann-Pick disease type C. *Orphanet J Rare Dis* 2010, 5:16.
3. Stampfer M, Theiss S, Amraoui Y, Jiang X, Keller S, Ory DS, Mengel E, Fischer C, Runz H. Niemann-Pick disease type C clinical database: cognitive and coordination deficits are early disease indicators. *Orphanet J Rare Dis* 2013, 8:35.
4. Wraith JE, Sedel F, Pineda M, Wijburg FA, Hendriksz CJ, Fahey M, Walterfang M, Patterson MC, Chadha-Boreham H, Kolb SA. Niemann-Pick type C Suspicion Index tool: analyses by age and association of manifestations. *J Inherit Metab Dis* 2014, 37(1):93-101.
5. Geberhiwot T, Moro A, Dardis A, Ramaswami U, Sirrs S, Marfa MP, Vanier MT, Walterfang M, Bolton S, Dawson C *et al*. Consensus clinical management guidelines for Niemann-Pick disease type C. *Orphanet J Rare Dis* 2018, 13(1):50.
6. Patterson MC, Hendriksz CJ, Walterfang M, Sedel F, Vanier MT, Wijburg F, Group N-CGW. Recommendations for the diagnosis and management of Niemann-Pick disease type C: an update. *Mol Genet Metab* 2012, 106(3):330-344.
7. Dardis A, Zampieri S, Gellera C, Carrozzo R, Cattarossi S, Peruzzo P, Dariol R, Sechi A, Deodato F, Caccia C *et al*. Molecular Genetics of Niemann-Pick Type C Disease in Italy: An Update on 105 Patients and Description of 18 NPC1 Novel Variants. *J Clin Med* 2020, 9(3).
8. Park WD, O'Brien JF, Lundquist PA, Kraft DL, Vockley CW, Karnes PS, Patterson MC, Snow K. Identification of 58 novel mutations in Niemann-Pick disease type C: correlation with biochemical phenotype and importance of PTC1-like domains in NPC1. *Hum Mutat* 2003, 22(4):313-325.
9. Higgins ME, Davies JP, Chen FW, Ioannou YA. Niemann-Pick C1 is a late endosome-resident protein that transiently associates with lysosomes and the trans-Golgi network. *Mol Genet Metab* 1999, 68(1):1-13.
10. Xu Y, Zhang Q, Tan L, Xie X, Zhao Y. The characteristics and biological significance of NPC2: Mutation and disease. *Mutat Res Rev Mutat Res* 2019, 782:108284.
11. Sleat DE, Wiseman JA, El-Banna M, Price SM, Verot L, Shen MM, Tint GS, Vanier MT, Walkley SU, Lobel P. Genetic evidence for nonredundant functional cooperativity between NPC1 and NPC2 in lipid transport. *Proc Natl Acad Sci U S A* 2004, 101(16):5886-5891.
12. Solomon BI, Smith AC, Sinaii N, Farhat N, King MC, Machielse L, Porter FD. Association of Miglustat With Swallowing Outcomes in Niemann-Pick Disease, Type C1. *JAMA Neurol* 2020, 77(12):1564-1568.
13. Grogan K. FDA rejects Actelion's Zavesca for rare NP-C disease. In.: PharmaTimes Online; 2010.
14. Actelion Pharmaceuticals Ltd (CH) - Actelion receives FDA complete response letter for Zavesca (miglustat) for the treatment of Niemann-Pick type C disease [https://www.drugs.com/nda/actelion-pharmaceuticals-ltd-ch-actelion-receives-fda-complete-response-letter-zavesca-miglustat-2828.html]
15. Bremova-Ertl T, Claassen J, Foltan T, Gascon-Bayarri J, Gissen P, Hahn A, Hassan A, Hennig A, Jones SA, Kolnikova M *et al*. Efficacy and safety of N-acetyl-L-leucine in Niemann-Pick disease type C. *J Neurol* 2022, 269(3):1651-1662.
16. Mengel E, Patterson MC, Da Riol RM, Del Toro M, Deodato F, Gautschi M, Grunewald S, Gronborg S, Harmatz P, Heron B *et al*. Efficacy and safety of arimoclomol in Niemann-Pick disease type C: Results from a double-blind, randomised, placebo-controlled, multinational phase 2/3 trial of a novel treatment. *J Inherit Metab Dis* 2021, 44(6):1463-1480.

17. Bremova-Ertl T, Ramaswami U, Brands M, Foltan T, Gautschi M, Gissen P, Gowing F, Hahn A, Jones S, Kay R *et al*. Trial of N-Acetyl-l-Leucine in Niemann-Pick Disease Type C. *N Engl J Med* 2024, 390(5):421-431.
18. Sharma R, Hastings C, Staretz-Chacham O, Raiman J, Paucar M, Spiegel R, Murray B, Hurst B, Liu B, Kjems L *et al*. Long-term administration of intravenous Trappsol(R) Cyclo (HP-beta-CD) results in clinical benefits and stabilization or slowing of disease progression in patients with Niemann-Pick disease type C1: Results of an international 48-week Phase I/II trial. *Mol Genet Metab Rep* 2023, 36:100988.
19. Ory DS, Ottinger EA, Farhat NY, King KA, Jiang X, Weissfeld L, Berry-Kravis E, Davidson CD, Bianconi S, Keener LA *et al*. Intrathecal 2-hydroxypropyl-beta-cyclodextrin decreases neurological disease progression in Niemann-Pick disease, type C1: a non-randomised, open-label, phase 1-2 trial. *Lancet* 2017, 390(10104):1758-1768.
20. Cagney DN, Sul J, Huang RY, Ligon KL, Wen PY, Alexander BM. The FDA NIH Biomarkers, EndpointS, and other Tools (BEST) resource in neuro-oncology. *Neuro Oncol* 2018, 20(9):1162-1172.
21. Walkley SU, Suzuki K. Consequences of NPC1 and NPC2 loss of function in mammalian neurons. *Biochim Biophys Acta* 2004, 1685(1-3):48-62.
22. Vig PJ, Subramony SH, Burright EN, Fratkin JD, McDaniel DO, Desaiah D, Qin Z. Reduced immunoreactivity to calcium-binding proteins in Purkinje cells precedes onset of ataxia in spinocerebellar ataxia-1 transgenic mice. *Neurology* 1998, 50(1):106-113.
23. Barski JJ, Hartmann J, Rose CR, Hoebeek F, Morl K, Noll-Hussong M, De Zeeuw CI, Konnerth A, Meyer M. Calbindin in cerebellar Purkinje cells is a critical determinant of the precision of motor coordination. *J Neurosci* 2003, 23(8):3469-3477.
24. Ko DC, Milenkovic L, Beier SM, Manuel H, Buchanan J, Scott MP. Cell-autonomous death of cerebellar purkinje neurons with autophagy in Niemann-Pick type C disease. *PLoS Genet* 2005, 1(1):81-95.
25. Vite CH, Bagel JH, Swain GP, Prociuk M, Sikora TU, Stein VM, O'Donnell P, Ruane T, Ward S, Crooks A *et al*. Intracisternal cyclodextrin prevents cerebellar dysfunction and Purkinje cell death in feline Niemann-Pick type C1 disease. *Sci Transl Med* 2015, 7(276):276ra226.
26. Kiyosawa K, Mokuno K, Murakami N, Yasuda T, Kume A, Hashizume Y, Takahashi A, Kato K. Cerebrospinal fluid 28-kDa calbindin-D as a possible marker for Purkinje cell damage. *J Neurol Sci* 1993, 118(1):29-33.
27. Campbell K, Cawley NX, Luke R, Scott KEJ, Johnson N, Farhat NY, Alexander D, Wassif CA, Li W, Cologna SM *et al*. Identification of cerebral spinal fluid protein biomarkers in Niemann-Pick disease, type C1. *Biomark Res* 2023, 11(1):14.
28. Baimbridge KG, Miller JJ, Parkes CO. Calcium-binding protein distribution in the rat brain. *Brain Res* 1982, 239(2):519-525.
29. Bradbury A, Bagel J, Sampson M, Farhat N, Ding W, Swain G, Prociuk M, O'Donnell P, Drobatz K, Gurda B *et al*. Cerebrospinal Fluid Calbindin D Concentration as a Biomarker of Cerebellar Disease Progression in Niemann-Pick Type C1 Disease. *J Pharmacol Exp Ther* 2016, 358(2):254-261.
30. Disanto G, Barro C, Benkert P, Naegelin Y, Schadelin S, Giardiello A, Zecca C, Blennow K, Zetterberg H, Leppert D *et al*. Serum Neurofilament light: A biomarker of neuronal damage in multiple sclerosis. *Ann Neurol* 2017, 81(6):857-870.
31. Kuhle J, Plattner K, Bestwick JP, Lindberg RL, Ramagopalan SV, Norgren N, Nissim A, Malaspina A, Leppert D, Giovannoni G *et al*. A comparative study of CSF neurofilament light and heavy chain protein in MS. *Mult Scler* 2013, 19(12):1597-1603.

32. Yuan A, Rao MV, Veeranna, Nixon RA. Neurofilaments at a glance. *J Cell Sci* 2012, 125(Pt 14):3257-3263.
33. Yin X, Crawford TO, Griffin JW, Tu P, Lee VM, Li C, Roder J, Trapp BD. Myelin-associated glycoprotein is a myelin signal that modulates the caliber of myelinated axons. *J Neurosci* 1998, 18(6):1953-1962.
34. Tortelli R, Ruggieri M, Cortese R, D'Errico E, Capozzo R, Leo A, Mastrapasqua M, Zoccolella S, Leante R, Livrea P *et al*. Elevated cerebrospinal fluid neurofilament light levels in patients with amyotrophic lateral sclerosis: a possible marker of disease severity and progression. *Eur J Neurol* 2012, 19(12):1561-1567.
35. Verde F, Steinacker P, Weishaupt JH, Kassubek J, Oeckl P, Halbgebauer S, Tumani H, von Arnim CAF, Dorst J, Feneberg E *et al*. Neurofilament light chain in serum for the diagnosis of amyotrophic lateral sclerosis. *J Neurol Neurosurg Psychiatry* 2019, 90(2):157-164.
36. Rosengren LE, Karlsson JE, Karlsson JO, Persson LI, Wikkelso C. Patients with amyotrophic lateral sclerosis and other neurodegenerative diseases have increased levels of neurofilament protein in CSF. *J Neurochem* 1996, 67(5):2013-2018.
37. Preische O, Schultz SA, Apel A, Kuhle J, Kaeser SA, Barro C, Graber S, Kuder-Buletta E, LaFougere C, Laske C *et al*. Serum neurofilament dynamics predicts neurodegeneration and clinical progression in presymptomatic Alzheimer's disease. *Nat Med* 2019, 25(2):277-283.
38. Norgren N, Rosengren L, Stigbrand T. Elevated neurofilament levels in neurological diseases. *Brain Res* 2003, 987(1):25-31.
39. Mattsson N, Andreasson U, Zetterberg H, Blennow K, Alzheimer's Disease Neuroimaging I. Association of Plasma Neurofilament Light With Neurodegeneration in Patients With Alzheimer Disease. *JAMA Neurol* 2017, 74(5):557-566.
40. Gaetani L, Blennow K, Calabresi P, Di Filippo M, Parnetti L, Zetterberg H. Neurofilament light chain as a biomarker in neurological disorders. *J Neurol Neurosurg Psychiatry* 2019, 90(8):870-881.
41. Gisslen M, Price RW, Andreasson U, Norgren N, Nilsson S, Hagberg L, Fuchs D, Spudich S, Blennow K, Zetterberg H. Plasma Concentration of the Neurofilament Light Protein (NFL) is a Biomarker of CNS Injury in HIV Infection: A Cross-Sectional Study. *EBioMedicine* 2016, 3:135-140.
42. Eratne D, Loi SM, Li QX, Varghese S, McGlade A, Collins S, Masters CL, Velakoulis D, Walterfang M. Cerebrospinal fluid neurofilament light chain is elevated in Niemann-Pick type C compared to psychiatric disorders and healthy controls and may be a marker of treatment response. *Aust N Z J Psychiatry* 2020, 54(6):648-649.
43. Agrawal N, Farhat NY, Sinaii N, Do AD, Xiao C, Berry-Kravis E, Bianconi S, Masvekar R, Bielekova B, Solomon B *et al*. Neurofilament light chain in cerebrospinal fluid as a novel biomarker in evaluating both clinical severity and therapeutic response in Niemann-Pick disease type C1. *Genet Med* 2023, 25(3):100349.
44. Dardis A, Pavan E, Fabris M, Da Riol RM, Sechi A, Fiumara A, Santoro L, Ormazabal M, Milanic R, Zampieri S *et al*. Plasma Neurofilament Light (NfL) in Patients Affected by Niemann-Pick Type C Disease (NPCD). *J Clin Med* 2021, 10(20).
45. Lund EG, Xie C, Kotti T, Turley SD, Dietschy JM, Russell DW. Knockout of the cholesterol 24-hydroxylase gene in mice reveals a brain-specific mechanism of cholesterol turnover. *J Biol Chem* 2003, 278(25):22980-22988.
46. Bjorkhem I, Cedazo-Minguez A, Leoni V, Meaney S. Oxysterols and neurodegenerative diseases. *Mol Aspects Med* 2009, 30(3):171-179.
47. Bjorkhem I. Crossing the barrier: oxysterols as cholesterol transporters and metabolic modulators in the brain. *J Intern Med* 2006, 260(6):493-508.

48. Bjorkhem I, Lutjohann D, Diczfalusy U, Stahle L, Ahlborg G, Wahren J. Cholesterol homeostasis in human brain: turnover of 24S-hydroxycholesterol and evidence for a cerebral origin of most of this oxysterol in the circulation. *J Lipid Res* 1998, 39(8):1594-1600.
49. Bielska AA, Olsen BN, Gale SE, Mydock-McGrane L, Krishnan K, Baker NA, Schlesinger PH, Covey DF, Ory DS. Side-chain oxysterols modulate cholesterol accessibility through membrane remodeling. *Biochemistry* 2014, 53(18):3042-3051.
50. Czuba E, Steliga A, Lietzau G, Kowianski P. Cholesterol as a modifying agent of the neurovascular unit structure and function under physiological and pathological conditions. *Metab Brain Dis* 2017, 32(4):935-948.
51. Janowski BA, Willy PJ, Devi TR, Falck JR, Mangelsdorf DJ. An oxysterol signalling pathway mediated by the nuclear receptor LXR alpha. *Nature* 1996, 383(6602):728-731.
52. Lehmann JM, Kliewer SA, Moore LB, Smith-Oliver TA, Oliver BB, Su JL, Sundseth SS, Winegar DA, Blanchard DE, Spencer TA *et al*. Activation of the nuclear receptor LXR by oxysterols defines a new hormone response pathway. *J Biol Chem* 1997, 272(6):3137-3140.
53. Abildayeva K, Jansen PJ, Hirsch-Reinshagen V, Bloks VW, Bakker AH, Ramaekers FC, de Vente J, Groen AK, Wellington CL, Kuipers F *et al*. 24(S)-hydroxycholesterol participates in a liver X receptor-controlled pathway in astrocytes that regulates apolipoprotein E-mediated cholesterol efflux. *J Biol Chem* 2006, 281(18):12799-12808.
54. Porter FD, Scherrer DE, Lanier MH, Langmade SJ, Molugu V, Gale SE, Olzeski D, Sidhu R, Dietzen DJ, Fu R *et al*. Cholesterol oxidation products are sensitive and specific blood-based biomarkers for Niemann-Pick C1 disease. *Sci Transl Med* 2010, 2(56):56ra81.
55. Beltroy EP, Richardson JA, Horton JD, Turley SD, Dietschy JM. Cholesterol accumulation and liver cell death in mice with Niemann-Pick type C disease. *Hepatology* 2005, 42(4):886-893.
56. Tortelli B, Fujiwara H, Bagel JH, Zhang J, Sidhu R, Jiang X, Yanjanin NM, Shankar RK, Carillo-Carasco N, Heiss J *et al*. Cholesterol homeostatic responses provide biomarkers for monitoring treatment for the neurodegenerative disease Niemann-Pick C1 (NPC1). *Hum Mol Genet* 2014, 23(22):6022-6033.
57. Maarup TJ, Chen AH, Porter FD, Farhat NY, Ory DS, Sidhu R, Jiang X, Dickson PI. Intrathecal 2-hydroxypropyl-beta-cyclodextrin in a single patient with Niemann-Pick C1. *Mol Genet Metab* 2015, 116(1-2):75-79.
58. Sidhu R, Jiang H, Farhat NY, Carrillo-Carrasco N, Woolery M, Ottinger E, Porter FD, Schaffer JE, Ory DS, Jiang X. A validated LC-MS/MS assay for quantification of 24(S)-hydroxycholesterol in plasma and cerebrospinal fluid. *J Lipid Res* 2015, 56(6):1222-1233.
59. Vanier MT, Gissen P, Bauer P, Coll MJ, Burlina A, Hendriksz CJ, Latour P, Goizet C, Welford RW, Marquardt T *et al*. Diagnostic tests for Niemann-Pick disease type C (NP-C): A critical review. *Mol Genet Metab* 2016, 118(4):244-254.
60. Jiang X, Ory DS. Advancing Diagnosis and Treatment of Niemann-Pick C disease through Biomarker Discovery. *Explor Neuroprotective Ther* 2021, 1(3):146-158.
61. Degtyareva AV, Proshlyakova TY, Gautier MS, Degtyarev DN, Kamenets EA, Baydakova GV, Rebrikov DV, Zakharova EY. Oxysterol/chitotriosidase based selective screening for Niemann-Pick type C in infantile cholestasis syndrome patients. *BMC Med Genet* 2019, 20(1):123.
62. Cooper JA, Church HJ, Wu HY. Cholestane-3beta, 5alpha, 6beta-triol: Further insights into the performance of this oxysterol in diagnosis of Niemann-Pick disease type C. *Mol Genet Metab* 2020, 130(1):77-86.
63. Sidhu R, Kell P, Dietzen DJ, Farhat NY, Do AND, Porter FD, Berry-Kravis E, Reunert J, Marquardt T, Giugliani R *et al*. Application of a glycinated bile acid biomarker for diagnosis and assessment of response to treatment in Niemann-pick disease type C1. *Mol Genet Metab* 2020, 131(4):405-417.

64. Mazzacuva F, Mills P, Mills K, Camuzeaux S, Gissen P, Nicoli ER, Wassif C, Te Vruchte D, Porter FD, Maekawa M *et al*. Identification of novel bile acids as biomarkers for the early diagnosis of Niemann-Pick C disease. *FEBS Lett* 2016, 590(11):1651-1662.
65. Hammerschmidt TG, Encarnacao M, Lamberty Faverzani J, de Fatima Lopes F, Poswar de Oliveira F, Fischinger Moura de Sousa C, Ribeiro I, Alves S, Giugliani R, Regla Vargas C. Molecular profile and peripheral markers of neurodegeneration in patients with Niemann-Pick type C: Decrease in Plasminogen Activator Inhibitor type 1 and Platelet-Derived Growth Factor type AA. *Arch Biochem Biophys* 2023, 735:109510.
66. Mandia D, Plaze M, Le Ber I, Ewenczyk C, Morin A, Carle G, Consoli A, Degardin A, Amad A, Moreau C *et al*. High diagnostic value of plasma Niemann-Pick type C biomarkers in adults with selected neurological and/or psychiatric disorders. *J Neurol* 2020, 267(11):3371-3377.
67. Jiang X, Sidhu R, Mydock-McGrane L, Hsu FF, Covey DF, Scherrer DE, Earley B, Gale SE, Farhat NY, Porter FD *et al*. Development of a bile acid-based newborn screen for Niemann-Pick disease type C. *Sci Transl Med* 2016, 8(337):337ra363.
68. Nunan J, Small DH. Regulation of APP cleavage by alpha-, beta- and gamma-secretases. *FEBS Lett* 2000, 483(1):6-10.
69. Selkoe DJ. Translating cell biology into therapeutic advances in Alzheimer's disease. *Nature* 1999, 399(6738 Suppl):A23-31.
70. Schoonenboom NS, Mulder C, Van Kamp GJ, Mehta SP, Scheltens P, Blankenstein MA, Mehta PD. Amyloid beta 38, 40, and 42 species in cerebrospinal fluid: more of the same? *Ann Neurol* 2005, 58(1):139-142.
71. Wiltfang J, Esselmann H, Bibl M, Smirnov A, Otto M, Paul S, Schmidt B, Klafki HW, Maler M, Dyrks T *et al*. Highly conserved and disease-specific patterns of carboxyterminally truncated Abeta peptides 1-37/38/39 in addition to 1-40/42 in Alzheimer's disease and in patients with chronic neuroinflammation. *J Neurochem* 2002, 81(3):481-496.
72. McLean CA, Cherny RA, Fraser FW, Fuller SJ, Smith MJ, Beyreuther K, Bush AI, Masters CL. Soluble pool of Abeta amyloid as a determinant of severity of neurodegeneration in Alzheimer's disease. *Ann Neurol* 1999, 46(6):860-866.
73. Xu H, Sweeney D, Wang R, Thinakaran G, Lo AC, Sisodia SS, Greengard P, Gandy S. Generation of Alzheimer beta-amyloid protein in the trans-Golgi network in the apparent absence of vesicle formation. *Proc Natl Acad Sci U S A* 1997, 94(8):3748-3752.
74. Howland DS, Trusko SP, Savage MJ, Reaume AG, Lang DM, Hirsch JD, Maeda N, Siman R, Greenberg BD, Scott RW *et al*. Modulation of secreted beta-amyloid precursor protein and amyloid beta-peptide in brain by cholesterol. *J Biol Chem* 1998, 273(26):16576-16582.
75. Avdulov NA, Chochina SV, Igbavboa U, Warden CS, Vassiliev AV, Wood WG. Lipid binding to amyloid beta-peptide aggregates: preferential binding of cholesterol as compared with phosphatidylcholine and fatty acids. *J Neurochem* 1997, 69(4):1746-1752.
76. Yamazaki T, Chang TY, Haass C, Ihara Y. Accumulation and aggregation of amyloid beta-protein in late endosomes of Niemann-pick type C cells. *J Biol Chem* 2001, 276(6):4454-4460.
77. Mattsson N, Zetterberg H, Bianconi S, Yanjanin NM, Fu R, Mansson JE, Porter FD, Blennow K. Gamma-secretase-dependent amyloid-beta is increased in Niemann-Pick type C: a cross-sectional study. *Neurology* 2011, 76(4):366-372.
78. Jin LW, Shie FS, Maezawa I, Vincent I, Bird T. Intracellular accumulation of amyloidogenic fragments of amyloid-beta precursor protein in neurons with Niemann-Pick type C defects is associated with endosomal abnormalities. *Am J Pathol* 2004, 164(3):975-985.
79. Mattsson N, Olsson M, Gustavsson MK, Kosicek M, Malnar M, Mansson JE, Blomqvist M, Gobom J, Andreasson U, Brinkmalm G *et al*. Amyloid-beta metabolism in Niemann-Pick C disease models and patients. *Metab Brain Dis* 2012, 27(4):573-585.

80. Love S, Bridges LR, Case CP. Neurofibrillary tangles in Niemann-Pick disease type C. *Brain* 1995, 118 (Pt 1):119-129.
81. Ong WY, Kumar U, Switzer RC, Sidhu A, Suresh G, Hu CY, Patel SC. Neurodegeneration in Niemann-Pick type C disease mice. *Exp Brain Res* 2001, 141(2):218-231.
82. March PA, Thrall MA, Brown DE, Mitchell TW, Lowenthal AC, Walkley SU. GABAergic neuroaxonal dystrophy and other cytopathological alterations in feline Niemann-Pick disease type C. *Acta Neuropathol* 1997, 94(2):164-172.
83. Blennow K, Wallin A, Agren H, Spenger C, Siegfried J, Vanmechelen E. Tau protein in cerebrospinal fluid: a biochemical marker for axonal degeneration in Alzheimer disease? *Mol Chem Neuropathol* 1995, 26(3):231-245.
84. Giannakopoulos P, Herrmann FR, Bussiere T, Bouras C, Kovari E, Perl DP, Morrison JH, Gold G, Hof PR. Tangle and neuron numbers, but not amyloid load, predict cognitive status in Alzheimer's disease. *Neurology* 2003, 60(9):1495-1500.
85. Villemagne VL, Velakoulis D, Dore V, Bozinoski S, Masters CL, Rowe CC, Walterfang M. Imaging of tau deposits in adults with Niemann-Pick type C disease: a case-control study. *Eur J Nucl Med Mol Imaging* 2019, 46(5):1132-1138.
86. Bountouvi E, Giorgi M, Papadopoulou A, Blennow K, Bjorkhem I, Tsirouda M, Kanellakis S, Fryganas A, Spanou M, Georgaki I *et al*. Longitudinal Data in Patients with Niemann-Pick Type C Disease Under Combined High Intrathecal and Low Intravenous Dose of 2-hydroxylpropyl-beta-cyclodextrin. *Innov Clin Neurosci* 2021, 18(1-3):11-16.
87. Mattsson N, Zetterberg H, Bianconi S, Yanjanin NM, Fu R, Mansson JE, Porter FD, Blennow K. Miglustat treatment may reduce cerebrospinal fluid levels of the axonal degeneration marker tau in niemann-pick type C. *JIMD Rep* 2012, 3:45-52.
88. Hastings C, Liu B, Hurst B, Cox GF, Hrynkow S. Intravenous 2-hydroxypropyl-beta-cyclodextrin (Trappsol(R) Cyclo) demonstrates biological activity and impacts cholesterol metabolism in the central nervous system and peripheral tissues in adult subjects with Niemann-Pick Disease Type C1: Results of a phase 1 trial. *Mol Genet Metab* 2022, 137(4):309-319.
89. Cariati I, Masuelli L, Bei R, Tancredi V, Frank C, D'Arcangelo G. Neurodegeneration in Niemann-Pick Type C Disease: An Updated Review on Pharmacological and Non-Pharmacological Approaches to Counteract Brain and Cognitive Impairment. *Int J Mol Sci* 2021, 22(12).
90. Giese AK, Mascher H, Grittner U, Eichler S, Kramp G, Lukas J, te Vruchte D, Al Eisa N, Cortina-Borja M, Porter FD *et al*. A novel, highly sensitive and specific biomarker for Niemann-Pick type C1 disease. *Orphanet J Rare Dis* 2015, 10:78.
91. Maekawa M, Jinnoh I, Matsumoto Y, Narita A, Mashima R, Takahashi H, Iwahori A, Saigusa D, Fujii K, Abe A *et al*. Structural Determination of Lysosphingomyelin-509 and Discovery of Novel Class Lipids from Patients with Niemann-Pick Disease Type C. *Int J Mol Sci* 2019, 20(20).
92. Sidhu R, Mondjinou Y, Qian M, Song H, Kumar AB, Hong X, Hsu FF, Dietzen DJ, Yanjanin NM, Porter FD *et al*. N-acyl-O-phosphocholineserines: structures of a novel class of lipids that are biomarkers for Niemann-Pick C1 disease. *J Lipid Res* 2019, 60(8):1410-1424.
93. Mashima R, Maekawa M, Narita A, Okuyama T, Mano N. Elevation of plasma lysosphingomyelin-509 and urinary bile acid metabolite in Niemann-Pick disease type C-affected individuals. *Mol Genet Metab Rep* 2018, 15:90-95.
94. Breilyn MS, Zhang W, Yu C, Wasserstein MP. Plasma lyso-sphingomyelin levels are positively associated with clinical severity in acid sphingomyelinase deficiency. *Mol Genet Metab Rep* 2021, 28:100780.
95. Cawley NX, Giddens S, Farhat NM, Luke RA, Scott KEJ, Mohamed HO, Dang Do A, Berry-Kravis E, Cologna SM, Liu F *et al*. Elevated cerebrospinal fluid ubiquitin C-terminal hydrolase-L1 levels

correlate with phenotypic severity and therapeutic response in Niemann-Pick disease, type C1. *Mol Genet Metab* 2023, 140(3):107656.

96. U.S. Food and Drug Administration. Biomarker Qualification: Evidentiary Framework Guidance for Industry and FDA Staff. In.; 2018.

97. Liao G, Yao Y, Liu J, Yu Z, Cheung S, Xie A, Liang X, Bi X. Cholesterol accumulation is associated with lysosomal dysfunction and autophagic stress in Npc1 -/- mouse brain. *Am J Pathol* 2007, 171(3):962-975.

98. Romanello M, Zampieri S, Bortolotti N, Deroma L, Sechi A, Fiumara A, Parini R, Borroni B, Brancati F, Bruni A *et al*. Comprehensive Evaluation of Plasma 7-Ketocholesterol and Cholestan-3beta,5alpha,6beta-Triol in an Italian Cohort of Patients Affected by Niemann-Pick Disease due to NPC1 and SMPD1 Mutations. *Clin Chim Acta* 2016, 455:39-45.

99. Reunert J, Lotz-Havla AS, Polo G, Kannenberg F, Fobker M, Griese M, Mengel E, Muntau AC, Schnabel P, Sommerburg O *et al*. Niemann-Pick Type C-2 Disease: Identification by Analysis of Plasma Cholestane-3beta,5alpha,6beta-Triol and Further Insight into the Clinical Phenotype. *JIMD Rep* 2015, 23:17-26.

100. Polo G, Burlina A, Furlan F, Kolamunnage T, Cananzi M, Giordano L, Zaninotto M, Plebani M, Burlina A. High level of oxysterols in neonatal cholestasis: a pitfall in analysis of biochemical markers for Niemann-Pick type C disease. *Clin Chem Lab Med* 2016, 54(7):1221-1229.

101. Hansson O, Kumar A, Janelidze S, Stomrud E, Insel PS, Blennow K, Zetterberg H, Fauman E, Hedman AK, Nagle MW *et al*. The genetic regulation of protein expression in cerebrospinal fluid. *EMBO Mol Med* 2023, 15(1):e16359.

102. Chiba Y, Komori H, Takei S, Hasegawa-Ishii S, Kawamura N, Adachi K, Nanba E, Hosokawa M, Enokido Y, Kouchi Z *et al*. Niemann-Pick disease type C1 predominantly involving the frontotemporal region, with cortical and brainstem Lewy bodies: an autopsy case. *Neuropathology* 2014, 34(1):49-57.

103. Ott BR, Cohen RA, Gongvatana A, Okonkwo OC, Johanson CE, Stopa EG, Donahue JE, Silverberg GD, Alzheimer's Disease Neuroimaging I. Brain ventricular volume and cerebrospinal fluid biomarkers of Alzheimer's disease. *J Alzheimers Dis* 2010, 20(2):647-657.

104. Albert OK, Friedmann K, Jaeger R, Berry-Kravis E. Low Risk Profile of Long-Term Repeated Lumbar Puncture for Intrathecal Delivery of 2-Hydroxypropyl-Beta-Cyclodextrin in Patients With Niemann-Pick Type C. *Pediatr Neurol* 2023, 144:99-103.

105. Rodriguez Ciancio JIR, Aquilina K. Complications associated with intrathecal drug delivery in a paediatric patient with Niemann-Pick type C. *BMJ Case Rep* 2021, 14(5).

106. Farhat NY, Farmer C, Do AD, Bianconi S, Porter FD. Low Incidence of Postdural Puncture Headache Further Reduced With Atraumatic Spinal Needle: A Retrospective Cohort Study. *Pediatr Neurol* 2021, 114:35-39.

107. U.S. Food and Drug Administration. Slowly Progressive, Low-Prevalence Rare Diseases With Substrate Deposition That Result From Single Enzyme Defects: Providing Evidence of Effectiveness for Replacement or Corrective Therapies Guidance for Industry. 2020.

Figure 1. Potential plasma and CSF biomarkers for neurodegeneration in Neimann-Pick Disease Type C (NPC). A) This figure depicts a schematic of healthy and NPC affected brain where neurofilament light chain (NfL), calbindin D, c-triol (cholestane-3β, 5a, 6β-triol), and amyloid-β (Aβ) are elevated in the cerebral spinal fluid (CSF) and 24(S)-hydroxycholesterol is reduced in the CSF. Similar trends in biomarkers are seen in plasma. B) In NPC, neurons may have neurofibrillary tangles of total and phosphorylated tau in the cell body of the neuron and are shown in dark red and purple. In the extracellular space, soluble and insoluble amyloid-β (Aβ) aggregates near the dendrites. Upon axonal injury, the highly expressed NfL in myelinated axons is leaked out into the interstitial fluid of the brain and in the CSF. Due to abberant cholesterol trafficking, conversion to 24(s)hydroxycholesterol is reduced due to a low supply of cholesterol as a substrate. Lastly, calbindin D may become elevated in the soma and intitial dendritic segments of the neuron potentially due to loss of cell loss. Created with BioRender.com.

Table 1. Current Biomarkers and Studies

Biomarker	Studies and Trials*	Findings	References
Calbindin D	Ory et al. (2017):<br>HPβCD in dose ranges from 50-1200 mg administered intrathecally monthly for 12 to 18 months in 14 patients with NPC and every 2 weeks for 18 months in 2 patients with NPC	Reduction in CSF concentrations at 12 or 18 months (statistically significant)	[19]
	NCT00344331:<br>Miglustat 200 mg TID (adjusted for body surface area in children) administered orally in patients with NPC	No change in CSF concentration between groups<br>Reduction in CSF concentration at 6-15 months between pre- and post-treatment (statistically significant)	[29]
	Bradbury et al. (2016): (feline model)<br>HPβCD 120 mg administered intrathecally every 2 weeks and were compared to untreated cats	*Reduction in CSF concentration (statistically significant)*	*[29]*
NfL	Agrawal et al. (2023):<br>HPβCD at 200, 300, 400, and 900 mg administered intrathecally (NCT01747135) and HPβCD at 900-1800 mg administered intrathecally every 2 weeks or a sham control (NCT02534844), or patients on miglustat (expanded access program part of IND119856)	Reduction in CSF concentrations with miglustat (statistically significant)<br><br>No change in CSF concentrations with HPβCD	[43]
24(S)-hydroxycholesterol	NCT01747135:<br>HPβCD 50 mg was administered into the lateral intraventricular space via Ommaya reservoir over 5 minutes and serial blood samples were collected pre-dose and 15, 30, 60 minutes and 3, 6, 24-, 36-, 48-, and 72-hours post-dose in an open-label trial	Increase in plasma concentration (statistically significant)	[56]
	Ory et al. (2017):<br>HPβCD in dose ranges from 50-1200 mg administered intrathecally monthly for 12 to 18 months in 14 patients with NPC and every 2 weeks for 18 months in 2 patients with NPC	Increase in the plasma concentration 72 hours after 900 and 1200 mg (statistically significant)<br><br>Increase in CSF concentration 72 hours after 600-900 mg (statistically significant)	[19]
	NCT02939547:<br>HPβCD 1500 mg/kg or 2500 mg/kg body weight administered intravenously every 2 weeks for 14 weeks (7 total infusions) in adult	Increase in serum concentration at Week 1, Day 2, and Week 12, Day 3 (no statistics)	[88]

	patients with NPC in a randomized, double-blind, parallel group design		
	Tortelli et al. (2014): (feline model) HPβCD 3 mg, 30 mg, and 120 mg or saline administered intrathecally to NPC1 cats aged 3 weeks to 11 weeks old	*Increase in plasma concentration at Day 3 (statistically significant) but not at other administrations*<br><br>*Increase in CSF concentration at Day 3 (statistically significant) Inconsistent CSF concentrations at other subsequent doses*	*[56]*
	Tortelli et al. (2014): (mouse model) HPβCD 6 mg/kg or saline was administered intracerebrovascularly followed by repeat administration 2 weeks later with 6 mg/kg or saline in Npc-/- mice	*Inconsistent results in plasma and CSF concentrations*	*[56]*
C-triol	NCT02612129:<br>Arimoclomol 93 to 372 mg/day TID or placebo administered orally for 12 months in patients with NPC aged 2 to 18 years (randomized 2:1)	Reduction in serum concentration at 12 months (not statistically significant)	[16]
	Hammerschmidt et al. (2023):<br>Miglustat 200 mg TID for 6 to 12 months	Reduction in plasma concentration at 12 months (statistically significant)	[65]
	NCT01747135:<br>HPβCD 50 mg was administered into the lateral intraventricular space via Ommaya reservoir over 5 minutes and serial blood samples were collected pre-dose and 15, 30, 60 minutes and 3, 6, 24-, 36-, 48-, and 72-hours post-dose in an open-label trial	Reduction in plasma concentrations by in two subjects after 25 and 24 months (statistically significant)	[56]
	Tortelli et al. (2014): (feline model) HPβCD 3 mg, 30 mg, and 120 mg or saline administered intrathecally to NPC1 cats aged 3 weeks to 11 weeks old	*Increase in plasma concentrations increased at 120 mg but not 3 or 30 mg (statistically significant)*	*[56]*
	Tortelli et al. (2014): (mouse model) HPβCD 6 mg/kg or saline was administered intracerebrovascularly followed by repeat administration 2 weeks later with 6 mg/kg or saline in Npc-/- mice	*Reduction in plasma concentrations after the first administration of and saline as the second (statistically significant)*<br><br>*Reduction in CSF concentrations after HPβCD as either the first or second administration and not repeat (statistically significant)*	*[56]*

TCG	NCT01747135: HPβCD 50-1200 mg was administered intrathecally every other week	Reduction in plasma concentrations after end of study (statistically significant)	[63]
	NCT02939547: three patients received HPβCD 500 or 2000 mg/kg administered intravenously every week	Reduction in plasma TCG concentrations (no statistics)	[63]
	NCT00344331: nine patients received 200 mg three times daily miglustat adjusted by body surface area	No change in plasma TCG	[29, 63]
Amyloid-β	Mattsson et al. (2012):<br>HPβCD 100 mg/kg and increased every dose until reaching 2,500 mg/kg administered intravenously in two patients with NPC and HPβCD 175 mg every 2 weeks administered intrathecally in addition to 2,500 mg/kg every 2 weeks administered intravenously in combination with oral miglustat	Reduction in CSF concentrations receiving miglustat and HPβCD (statistically significant)	[79]
	Mattsson et al. (2012): (feline model) HPβCD 1000 mg/kg body weight, 4000 mg/kg body weight or 8,000 mg/kg body weight administered subcutaneously weekly, or 4000 mg/kg brain weight administered intrathecally every other week to NPC cats compared to untreated cats	*Reduction in CSF concentration of Aβ37, Aβ38, and Aβ39 following HPβCD; Increase in CSF concentration of Aβ1-16 and Aβ28 (no statistics)*	*[79]*
Total and phosphorylated tau	NCT02939547:<br>HPβCD 1500 mg/kg or 2500 mg/kg body weight administered intravenously every 2 weeks for 14 weeks (7 total infusions) in adult patients with NPC in a randomized, double-blind, parallel group design	Reduction in CSF concentrations (not statistically significant)	[88]
	Mattsson et al. (2012):<br>HPβCD 100 mg/kg and increased every dose until reaching 2,500 mg/kg administered intravenously in two patients with NPC and HPβCD 175 mg every 2 weeks administered intrathecally in addition to 2,500 mg/kg every 2 weeks administered intravenously in combination with oral miglustat	Reduction in CSF concentrations (statistically significant)	[79]
PPCS	NCT02612129:<br>Arimoclomol 93 to 372 mg/day TID or placebo administered orally for 12 months in patients with NPC aged 2 to 18 years (randomized 2:1)	Reduction in plasma concentrations at 6- and 12-months following treatment (statistically significant)	[16]

	NCT02939547: HPβCD 1500 mg/kg or 2500 mg/kg body weight administered intravenously every 2 weeks for 14 weeks (7 total infusions) in adult patients with NPC in a randomized, double-blind, parallel group design	Reduction in plasma concentrations at Week 14 (no statistics)	[88]

Aβ, amyloid β; C-triol, cholestane 3β, 5α, 6β-triol; CSF, cerebral spinal fluid; HPβCD, 2-hydroxypropyl-β-cyclodextrin; IV, intravenous; PPCS, N-palmitoyl-O-phosphocholine-serine; NfL, neurofilament light chain; NPC, Niemann-Pick Disease Type C; TCG, TID, three times a day;

*Findings as reported in literature, animal studies are italicized
