# Supplementary material for: Evaluation of the landscape of pharmacodynamic biomarkers in Niemann-Pick Disease Type C (NPC)
Source: Orphanet J Rare Dis. 2024 Jul 26;19:280. doi: 10.1186/s13023-024-03233-7 (PMC11282650; doi:10.1186/s13023-024-03233-7)
Supplement: Supplementary file 2 — Supplementary Material 2 [file 13023_2024_3233_MOESM2_ESM.docx]

BMC Orphanet	May 20, 2024

Re: manuscript OJRD-D-23-01029

Dear Dr.Pode-Shakked,

Enclosed please find our second revised manuscript titled “Evaluation of the Landscape of Pharmacodynamic Biomarkers in Niemann-Pick Disease Type C (NPC)” for consideration of publication in the Orphanet Journal of Rare Diseases.

We have revised the manuscript per reviewers’ second round of comments. The revised version of the manuscript with tracked changes is included. Our responses to reviewers’ comments are detailed and appended to this cover letter.

We look forward to hearing from you regarding our manuscript. Thank you for your kind assistance.

Best regards,

Sydney Stern

U.S. Food and Drug Administration

Office of Translational Science; Office of Clinical Pharmacology

Division of Translational and Precision Medicine

10903 New Hampshire Ave,

Silver Spring, MD 20993

email: Sydney.Stern@FDA.hhs.gov

**Reviewer #1:** Critiques adequately addressed

*We appreciate the reviewer’s comments which improved the manuscript.*

**Reviewer #2:**

The authors have clarified ambiguities, corrected errors, and improved readability, but the manuscript still feels wordy, and a bit repetitive. The detailed and exhaustive catalog of existing studies may be useful, but the authors tend to make multiple general and rather obvious observations at a considerable length ( e.g. "Identification of a biomarker with a sensitive method could enable prompt diagnosis and treatment with the goal of identifying effective therapy that could delay or prevent neurological manifestations." or " the current level of evidence supports further investigations into biomarkers for drug development in NPC, particularly demonstrating the biomarkers' relation to the natural history of the disease and supported by mechanistic understanding of how the biomarker is involved in the disease pathway. It is necessary to further characterize the utility and sensitivity of biomarkers to detect treatment response."). Such general statements should be avoided, after all, the article is intended, I assume, for readers with some knowledge of the field.

*We thank the reviewer for their feedback and have made every attempt to reduced generalized statements throughout the manuscript while acknowledging the need for more data which correlates biomarkers to clinical assessments and elucidates the role in the pathophysiology of NPC across all biomarkers.*

The authors suggest that "more comprehensive panels of biomarkers, rather than a single biomarker for all contexts of use and all drugs under investigation", but their evaluation of studies from that point of view is limited (it is available in the section on tau, for instance), even if multiple biomarkers were tested and discussed e.g. Ory et.al. Lancet 2017; 390: 1758-68.

*We have clarified the text to include that “it is our opinion that more comprehensive panel of biomarkers, rather than a single biomarker for all contexts of use and all drugs under investigation, are needed to robustly define and optimize patient response.” We have additionally added an example of where a combination of biomarkers may strengthen support for a treatment effect in lines 612-615 on pg 27.*

I believe that the manuscript could be further improved by focusing on key messages for each marker and removal of vague generalizations.

*We thank the reviewer and agree with highlighting key messages for each biomarker. We have included a summary sentence to highlight findings for each biomarker in the last paragraph of each section. We removed general repetitive statements throughout the manuscript and focused our discussion on specific considerations across biomarkers.*

**Reviewer #3:**

In most instances, the authors have modified their manuscript to comply to the points raised by reviewer 3 (sometimes with a minimal effort, but acceptable). Nevertheless, a few points still need revision.

*We appreciate the reviewer’s extensive and thoughtful comments to improve the manuscript.*

All three reviewers have required a rewriting of the "lysosphingomyelin-509" section, and correction to the proper name of this compound. The authors now properly refer to N-palmitoyl-O-phosphocholineserine (PPCS). But the text on lines 478-491 (on clean manuscript) should be considerably simplified and made clearer.

*We thank the reviewer for their feedback and have made every attempt to simplify the language and improve readability throughout the manuscript.*

Line 479: "screening and diagnosis of NPC have advanced to include composite biomarker panels which incorporate a class of novel lipids, namely PPCS" : this is slightly incorrect, PPCS is a specific compound -- N-palmitoyl-O-phosphocholineserine -- within a novel class of lipids, N-acyl-O-phosphochoineserines; this seems unclear to the authors.

*We have clarified the text as follows: “Screening and diagnosis of NPC incorporate a novel lipid known as N-palmitoyl-O-phosphocholine-serine (PPCS) [5, 60, 90].” in line 487-489 on pg. 22.*

Should rather be written as "Screening of NPC has advanced to also include in biomarker panels a compound belonging to a novel class of lipids, namely PPCS". Among cited references, delete [90], which is not dealing with lysosphingomyelin-509/PPCS.

*We have modified the text as previously described and removed the citation.*

Lines 481-491: Details related to elucidation of the correct structure of the compound do not belong to this review, and should be deleted. The authors should just indicate that elucidation of its structure has now been made as PPCS [93,92] (note that the paper by Sidhu et al was published several months earlier than that by Maekawa et al, so the order of the references should be corrected).

*We appreciate the reviewer’s feedback and have simplified the text.*

Details of PPCS use in screening panels seem somewhat out of the scope of this review, but this reviewer won't argue on that point.

*We agree with the reviewer that the adequacy of biomarkers in screening and diagnostics is out of scope of this review. However, we hope current knowledge can be leveraged to demonstrate the potential utility of PPCS as a treatment response biomarker by providing foundational understanding that there is a distinct elevation at baseline.*

Discussion, lines 526-629: the authors should clearly indicate that biomarkers, in combination with clinical symptoms, are good indicators of disease, but that a diagnosis of NPC further requires genetic testing (sequencing of the NPC1 and NPC2 genes, and sometimes more refined genetic analysis).

*We appreciate the reviewer bringing this to our attention and we agree that diagnostics are outside the scope of this review. We have removed the paragraph and refocused the discussion around biological plausibility of lipid biomarkers in NPC pathophysiology.*

Line 532: C-triol, TCG, PPCS are not specific for NPC; they are all elevated also in ASMD (and PPCS is now widely used for follow up of enzyme replacement with olipudase alfa).

*We agree with the reviewer and acknowledge the lack of specificity of these biomarkers to discern NPC from other diseases and have clarified the text to include that these biomarkers are not specific to NPC pathophysiology and future studies should assess how these biomarkers correspond to clinical assessment.*
